# Clinical performance, safety, and patient-reported outcomes of an active osseointegrated bone-conduction hearing implant system at 24-month follow-up

**DOI:** 10.1007/s00405-023-08133-3

**Published:** 2023-08-08

**Authors:** Robert Cowan, Aaran T. Lewis, Carina Hallberg, Michael C. F. Tong, Catherine S. Birman, Iris H.-Y. Ng, Robert Briggs

**Affiliations:** 1 Department of Otolaryngology, The University of Melbourne, Melbourne, Australia; 2 Cochlear Limited, Sydney, Australia; 3 Department of Otorhinolaryngology, Head and Neck Surgery and Institute of Human Communicative Research, The Chinese University of Hong Kong, Hong Kong SAR, People’s Republic of China; 4 Nextsense, Sydney, Australia; 5 Faculty of Medicine and Health, Sydney University, Sydney, Australia; 6 Faculty of Medicine and Health Sciences, Macquarie University, Sydney, Australia

**Keywords:** Active transcutaneous bone conduction implant, Conductive and mixed hearing loss, Semi-implantable hearing device, Single-sided deafness, Speech recognition in quiet, Speech recognition in noise

## Abstract

**Purpose:**

To investigate 2-year post-operative hearing performance, safety, and patient-reported outcomes of hearing-impaired adults treated with the Osia^®^ 2 System, an active osseointegrated bone-conduction hearing implant that uses piezoelectric technology.

**Methods:**

A prospective, multicenter, open-label, single-arm, within-subject clinical study conducted at three tertiary referral clinical centers located in Melbourne, Sydney and Hong Kong. Twenty adult recipients of the Osia 2 System were enrolled and followed up between 12 and 24 months post-implantation: 17 with mixed or conductive hearing loss and 3 with single-sided sensorineural deafness. Safety data, audiological thresholds, speech recognition thresholds in noise, and patient-reported outcomes were collected and evaluated. In addition, pre-and 6-month post-implantation data were collected retrospectively for this recipient cohort enrolled into the earlier study (ClinicalTrials.gov NCT04041700).

**Results:**

Between 6- and 24-month follow-up, there was no statistically significant change in free-field hearing thresholds or speech reception thresholds in noise (*p* =  > 0.05), indicating that aided improvements were maintained up to 24 months of follow-up. Furthermore, improvements in health-related quality of life and daily hearing ability, as well as clinical and subjective measures of hearing benefit remained stable over the 24-month period. No serious adverse events were reported during extended follow-up.

**Conclusions:**

These study results provide further evidence to support the longer term clinical safety, hearing performance, and patient-related benefits of the Osia 2 System in patients with either a conductive hearing loss, mixed hearing loss, or single-sided sensorineural deafness.

**Trial Registration:**

ClinicalTrials.gov Identifier: NCT04754477. First posted: February 15, 2021.

## Introduction

The Osia^®^ System is an active bone-conduction hearing implant indicated for patients with conductive hearing loss (CHL), mixed hearing loss (MHL), and single-sided deafness (SSD). The osseointegrated implant stimulates the skull bone directly using an implanted piezoelectric transducer and is controlled and powered by an external sound processor that captures external sounds via the microphones. A key advantage of the Osia System compared to passive devices is the gain provided at higher frequencies compared to passive bone conduction [1].

The system has a fitting range of up to 55 dB HL. An overview of the current system design and function and short-term clinical outcomes for a cohort of 29 subjects have been published previously [1]. The safety and efficacy of the Osia System has also been reported in several additional clinical studies [2–5]. However, no study has presented longer term (> 12 month) follow-up data with the current system to date. Therefore, the aim of this study was to evaluate the longer term safety and efficacy of the Osia 2 System through the collection of safety, audiological, and quality of life data up to 24 months post-implantation. As the device is expected to be implanted for many years, it is important to characterize the long-term safety profile and clinical benefits of the system.

## Methods

### Study information

This study collected 12- and 24-month follow-up data from the enrolled group of Osia System recipients, who had existing data from a previous study, at pre-implantation and 6 months post-implantation [1]. The current study was approved by respective local ethics committees as per local regulations, conducted in accordance with the Declaration of Helsinki [6] and ISO14155:2011[7] and was registered on ClinicalTrials.gov with identifier NCT04754477.

### Investigational device

The investigational device was the Cochlear™ Osia 2 System (Cochlear Ltd., Sydney, Australia) consisting of an external sound processor (SP) (Osia 2 SP) magnetically retained on the skin over the site of an internal implant (OSI200 Implant), which is fixed to the temporal bone with an osseointegrating implant (BI300 screw fixture, 3 or 4 mm). Surgical technique has been published previously [1] and the SP was individually fitted to each subject's hearing loss using Osia Fitting Software 2.0.

### Study schedule and assessments

The objective of the clinical study was to compare safety parameters, patient-reported outcomes (PRO), and change in free-field hearing thresholds and speech reception threshold (SRT) in noise (dB SNR) with the Osia 2 System at 6 months and 12 and 24 months post-implantation. As part of the current study, visits were carried out at 12 and 24 months after implantation with the Osia 2 System for efficacy and safety evaluation. Data collected between baseline (pre-implantation) and 6-month follow-up post-implantation with the Osia 2 System were retrospectively collected from the previous clinical study sponsored by Cochlear (ClinicalTrials.gov NCT04041700). Safety parameters were recorded throughout the current clinical study and retrospectively collected for enrolled patients in the previous clinical study between implantation with the Osia 2 System and 6-month follow-up.

### Hearing thresholds

The tests were performed in a sound-insulated audiometric booth using calibrated equipment with the non-test ear blocked using both a foam plug and earmuffs in case of normal or near-normal hearing or a large asymmetry between ears. During testing, SPs were set to fixed directionality mode. Threshold audiometry was performed using warble tones presented via a loudspeaker located 1 m in front of the subjects at 0-degree azimuth, with 1 m of free space surrounding the test subject. PTA4 hearing thresholds were calculated by taking an average of hearing thresholds measured at 0.5, 1, 2, and 4 kHz. Changes in audiometric thresholds of > 10 dB at a single frequency are considered as clinically relevant as outside of the test–retest variability [8].

### Speech recognition in noise

For speech in noise testing, both speech and noise were presented in free field at 0-degree azimuth (front). In Melbourne and Sydney, the AuSTIN test was administered and sentences were presented at a constant level of 65 dB SPL throughout the test, and babble noise was adapted stepwise according to the software used to establish the signal-to-noise ratio (SNR) providing a 50% level of correctly repeated morphemes. In Hong Kong, the CHINT methodology was used, where noise was maintained at a constant 65 dB SPL, and sentence speech stimuli were presented adaptatively, with stepwise adjustments to the speech level made by the software to establish the SNR where the test subject repeated 50% of the material correctly. Improvements of greater than 3 dB SNR are considered to be clinically relevant [9].

### Patient-reported outcomes

Patient-reported outcome measures were collected using validated questionnaires: the Health Utilities Index (HUI-3) [10, 11], the Abbreviated Profile of Hearing Aid Benefit (APHAB) [12, 13], and the Speech, Spatial and Qualities of Hearing Scale 12 (SSQ12) [14, 15]. HUI-3 evaluates eight health-related quality of life (QoL) dimensions (vision, hearing, speech, walking/mobility, dexterity, self-care, emotion, and cognition) and a comprehensive health state attribute. A change in global HUI-3 score of 0.03 or higher is considered clinically relevant [16]. APHAB is a hearing-related PRO instrument, which includes four subscales (ease of communication, reverberation, and background noise, aversiveness) and a global score. A change in score of higher than 10 for global score is generally regarded as clinically relevant [17]. SSQ12 is a short (12-item) version of the original 49-item SSQ questionnaire [18] that measures the self-reported auditory disability in everyday life across three subdomains (speech, spatial, and qualities of hearing). Changes of 1.0 unit or greater on SSQ subscales indicate a clinically relevant change [18]. Data between baseline and 6 months post-implantation were retrospectively collected from the previous clinical study. PRO at 12 and 24 months post-implantation were prospectively collected as part of the current study.

Patient-reported daily usage, wearing comfort, and retention were collected at all study visits after activation. Daily use communicated by the patient was reported as the average hours of daily SP use during the period preceding the visit. Comfort and retention were assessed subjectively by indicating on a visual analog scale consisting of a straight line running from 0 to 100 mm (0 mm indicated no comfort at all/insufficient retention, and 100 mm indicated the most comfortable situation imaginable/excellent retention).

### Statistical analysis

Analyses were performed on all subjects enrolled in the current study. All statistical analyses were paired and nonparametric and the Friedman’s test was used to test for paired observations. All significance tests were two-tailed and performed at the 0.05 significance level. All significance values were adjusted by the Bonferroni correction for multiple tests. Statistical analysis was performed in SAS version 9.4 (SAS Institute Inc.) and IBM SPSS Statistics, version 28 (Armonk, NY: IBM Corp.). Demographics, baseline characteristics, daily use, comfort, adverse events, and device deficiencies are presented descriptively.

## Results

The data of 20 subjects attending the 6-month follow-up visit in the previous study [1] who consented to participate in this extended follow-up study and attended the 12- or 24-month visit were combined and analyzed. Subject demographics can be seen in Table 1. As the study did not control for type of hearing loss, the majority had CHL or MHL (*N* = 17) and three with SSD. Data are presented as means and *p* values, where *p* values are presented for the sample as a whole.

**Table 1:** Patient demographics for the 20 subjects enrolled in the study

Variable	Total (*N* = 20)
Age (years)	44.9 (20.8)
Gender	
Male	9 (45.0%)
Female	11 (55.0%)
Type of hearing loss	
Single-sided deafness	3 (15.0%)
Conductive	11 (55.0%)
Mixed	6 (30.0%)
Aetiology (test side)	
Chronic otitis media	4 (20.0%)
Tumor	1 (5.0%)
Malformation	7 (35.0%)
Unknown	2 (10.0%)
Other	6 (30.0%)
History of hearing loss (test side)	
Progressive	7 (35.0%)
Progressive with sudden	2 (10.0%)
Sudden	2 (10.0%)
Congenital with progression	4 (20.0%)
Congenital without progression	5 (25.0%)

### Safety evaluation

**Fig. 1:**
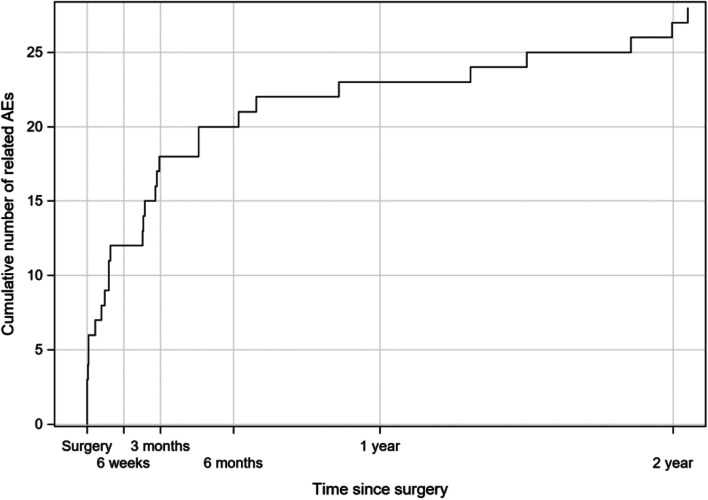
Cumulative number of related adverse events between implantation and 24-month follow-up (*N* = 20). *AEs* adverse events

Between 6- and 24-month follow-up, there were nine adverse events in six subjects that were either possibly, probably or definitely related to the device or procedure. Three of these events (pain behind implant, skin irritation, and prominence of the posterior inferior edge of system) were related to both device and procedure and six were related to the device only (discomfort from SP heating up, increased tinnitus, two reports of frustration, and two reports of non-use). The two reports of non-use were classified as moderate in severity and the remaining events were all classified as mild in severity. Figure 1 presents the cumulative adverse event rate for all related adverse events from implantation to 24-month follow-up. The majority of related adverse events occurred within the first 3 months post-implantation and the rate decreased between 3 and 24 months. All but three adverse events (two reports of non-use and one report of frustration) related to device or procedure reported in two subjects were resolved by study end.

### Hearing performance

**Fig. 2:**
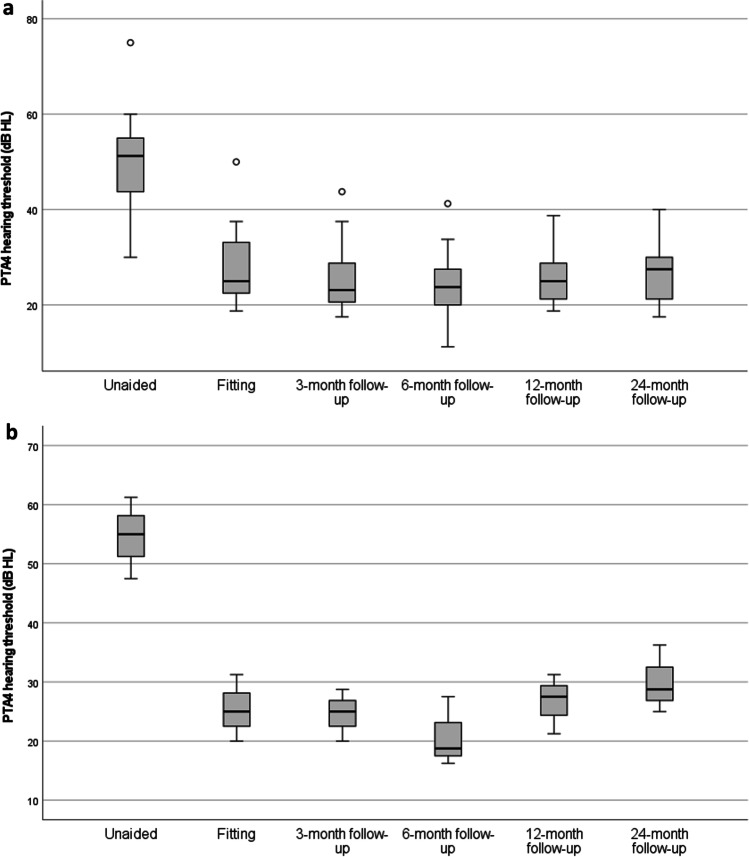
Box plot of the distribution of PTA4 hearing thresholds for subjects with MHL/CHL (*N* = 17) (**a**) and SSD (*N* = 3) (**b**) between the unaided situation and 24-month follow-up. Open circles represent outliers and lines within boxes represent the median values of each follow-up. The change between the unaided situation and all follow-ups is statistically significant for the cohort (*p* values < 0.05)

Mean change in pure-tone audiometry four frequency average (PTA4) from 6-months to 24-months follow-up was − 3.7 dB HL (SD: 5.2 dB HL, range − 5.0 to 13.8. dB HL), while the mean change in PTA4 from 6-months to the 12-month follow-up period was − 2.2 dB HL (SD: 5.1 dB HL, range − 6.3 to 13.8. dB HL). There were no statistically significant differences in paired comparisons between the 6-month follow-up and 24-month follow-up (all *p* values > 0.05), indicating stable improvements up to 24-month follow-up with the investigational device for subjects with MHL/CHL and those with SSD (Fig. 2). There were also statistically significant (all *p*-values < 0.001) improvements in PTA4 hearing thresholds between pre-implantation and all subsequent aided follow-up measurements. At the 12-month follow-up, the mean improvement in PTA4 from the unaided situation was 26.7 dB HL (SD: 7.0 dB HL, range − 43.8 to − 13.8 dB HL). At the 24-month follow-up, the mean improvement in PTA4 from the unaided situation was 25.5 dB HL (SD: 6.5 dB HL, range − 37.5 to − 12.5 dB HL).

**Fig. 3:**
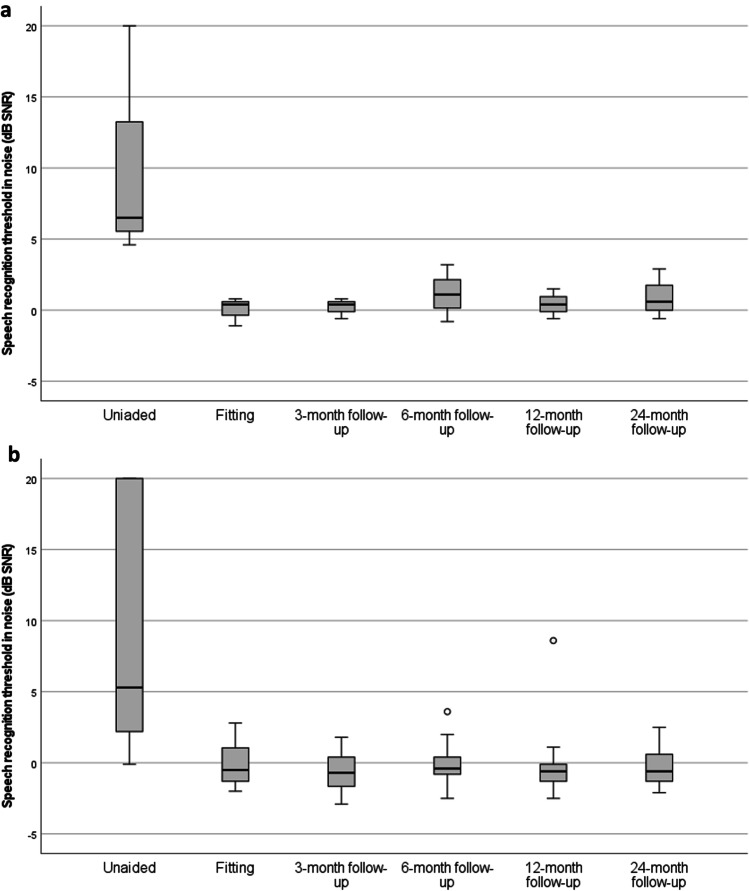
Box plot of the distribution of speech recognition thresholds for subjects with MHL/CHL (*N* = 17) (**a**) and SSD (*N* = 3) (**b**) between the unaided situation and 24-month follow-up. Open circles represent outliers and lines within boxes represent the median values of each follow-up. The change between the unaided situation and all follow-ups is statistically significant for the cohort (*p* values < 0.05)

For the adaptive speech recognition in noise test, there was no statistically significant difference when comparing the investigational device at the 12- and 24-month follow-up to the aided situation at the last performed assessment 6 months after surgery in the previous clinical study (all *p* values > 0.05). Mean change in SRT in noise from 6 months to the 24-month follow-up visit was -0.244 dB SNR (SD: 0.913 dB SNR, range − 1.7 to 1.4 dB SNR). While the mean change in SNR from 6-months to the 12-month follow-up period was − 0.106 dB SNR (SD: 2.726 dB SNR, range − 3.8 to 9.2 dB SNR). These results indicate stable improvements for speech recognition up to 24-month follow-up for subjects with MHL/CHL and SSD (Fig. 3). Compared to the unaided situation, the SRT in noise improved by 8.7 dB SNR (SD 7.2 dB, range − 22.5 to 0.0 dB) when aided with the Osia System at the 12-month follow-up. At the 24-month follow-up, the average improvement with the Osia System was 8.7 dB (SD: 7.2 dB, range − 21.3 to − 1.7 dB) compared to the unaided situation at baseline.

### Patient-reported outcomes

#### Health Utilities Index-Mark 3

When comparing the investigational device at the 12- and 24-month follow-up to the aided situation at the last performed assessment 6-month post-surgery in the previous study (Table 2), no statistically significant changes were seen for any of the single-attribute utility scores or for the multi-attribute utility score (all *p* values > 0.05), indicating stable outcomes between 6 and 24 months. There was also no statistically significant difference when comparing single-attribute scores or multi-attribute utility score with the investigational device between fitting and 24-month follow-up (all *p*-values > 0.05), indicating stable improvements in HUI-3 scores up to 24-months.

**Table 2:** Mean change in PROs from 6- to 12-month follow-up and from 6- to 24-month follow-up

Variable	Change from month 6 to 12, mean (SD; range)	Change from month 6 to 24, mean (SD; range)
HUI-3 multi-attribute utility	0.006 (0.115; − 0.195 to 0.275)	− 0.045 (0.151; − 0.410 to 0.174)
APHAB global	31.6 (21.7; − 2.8 to 69.6)	− 0.6 (9.5; − 30.5 to 13.6)
APHAB communication	− 0.5 (9.1; − 25.2 to 8.5)	0.9 (10.6; − 33.0 to 17.0)
APHAB background noise	1.6 (10.6; − 14.2 to 22.7)	0.1 (11.8; − 29.0 to 18.9)
APHAB reverberation	− 1.3 (13.9; − 34.8 to 16.8)	− 2.8 (14.7; − 29.5 to 27.2)
APHAB aversiveness	5.9 (15.3; − 16.8 to 35.0)	2.0 (18.0; − 35.0 to 41.5)
SSQ total	0.2 (0.8; − 1.5 to 1.9)	− 0.2 (1.4; − 4.0 to 2.5)
SSQ speech	0.4 (1.1; − 1.4 to 2.3)	0.2 (1.5; − 3.7 to 3.1)
SSQ spatial	0.4 (1.1; − 1.6 to 2.1)	− 0.2 (2.0; − 4.7 to 3.8)
SSQ qualities	− 0.1 (2.6; − 2.6 to 8.1)	− 0.6 (1.5; − 4.0 to 3.4)

^*SD* Standard Deviation, *HUI−3* Health Utilities Index Mark 3, *APHAB* Abbreviated Profile of Hearing Aid Benefit, *SSQ* Speech, Spatial and Qualities of Hearing Scale^

Compared to the unaided listening situation before implantation, the HUI-3 multi-attribute utility score improved by 0.080 (SD 0.179, range − 0.192 to 0.329, *p* = 0.067) at the 12-month follow-up and by 0.028 (SD: 0.237, range − 0.41 to 0.426, *p* = 0.610) at the 24-month follow-up visit. HUI-3 hearing attribute score improved by 0.159 (SD 0.309, range − 0.290 to 0.710, *p* = 0.040) at the 12-month follow-up with the investigational device compared to the unaided situation before implantation. At the 24-month follow-up, the mean improvement in the hearing attribute score was 0.126 (SD: 0.355, range − 0.680 to 0.710, *p* = . 150) compared to the unaided condition prior to Osia system implantation.

#### Abbreviated profile of hearing aid benefit

When comparing the investigational device at the 12- and 24-month follow-up to the aided situation at the last performed assessment at the 6-month follow-up in the previous study, no statistically significant changes were seen for any of the APHAB subscale scores (all *p* values > 0.05) (Table 2). There was also no statistically significant difference when comparing individual subdomain scores or global APHAB score with the investigational device between sound processor fitting and 24-month follow-up (all *p* values > 0.05) (Table 2). This indicated stable hearing status in all APHAB subscales up to 24-month follow-up with the investigational device.

#### The speech, spatial and qualities of hearing scale

When comparing the investigational device at the 12- and 24-month follow-up to the aided situation at the last performed assessment at 6 months in the previous study, no statistically significant mean changes were seen for any of the SSQ scores (all *p* values > 0.05) (Table 2), indicating stable improvements in SSQ scores up to 24-month follow-up with the investigational device for the study cohort. SSQ global score improved by 3.14 (SD 1.74, range − 0.11 to 6.26, *p* =  < 0.001) at the 12-month follow-up with the investigational device compared to the unaided situation, pre-implantation. At the 24-month follow-up, the mean improvement in global SSQ score was 2.53 compared to unaided listening before Osia system implantation (SD: 2.19, range − 1.13 to 6.78, *p* =  < 0.001).

#### Satisfaction, battery lifetime, and device usage

At 12 months post-implant, all subjects who completed the questionnaire (*N* = 19) reported that they were satisfied with the implanted device. At 24 months, 18/19 subjects were either satisfied or very satisfied with the investigational device overall. One subject with MHL/CHL who was not satisfied reported that they were unsatisfied with the sound quality of the system and had stopped wearing their device. The non-user with SSD reported that they were satisfied with their device.

Battery lifetime remained stable between the 6-month and the 24-month follow-up visits. Mean battery lifetime was 24.7 h (SD: 12.9 h) at the 6-month follow-up and 23.3 h (SD: 9.2 h) at the 24-month follow-up visit.

Daily device usage was consistent from 8.2 h (SD: 4.6 h) to 8.5 h (SD: 5.2) at the 6-month and 24-month follow-up visits, respectively. Retention ratings also remained stable between 6- and 24-month follow-up (75.7/100 at 6 months and 82.8/100 at 24 months). The device comfort rating also remained stable between 6- and 24-month follow-up (86.7/100 at 6 months and 87.8/100 at 24 months).

## Discussion

This cohort of adults implanted with the Osia 2 System was followed up over a 24-month period. Audiological outcomes, PROs and safety data collected throughout this extended follow-up study demonstrated that the implant system remains safe and that it continues to provide clinically important longer term aural habilitation/rehabilitation for subjects with MHL/CHL or SSD.

### Safety outcomes

The acceptable low rate of adverse events reported in this study is also in line with a recent evidence synthesis of adverse event rates for active bone-conduction hearing implant systems, including the Osia System [19]. Events reported during the 24-month follow-up (*N* = 19) were classified as mild or moderate. It was apparent that the rate of adverse events decreased markedly after the initial 3–6-month follow-up period post-implantation and that this continued to decrease with time. Rates of adverse events in studies with limited follow-up (i.e., ≤ 6 months) may therefore not be useful to model complication rates over the longer term and may lead to an overestimation of complication rates. These data can be used to inform counseling and future cost-effectiveness modeling with active bone-conduction hearing implants. Regarding the two subjects who had stopped using their device regularly, one retired subject with SSD did not feel the need for the Osia device due to social isolation during COVID-19 restrictions. Non-compliance rates with bone-conduction hearing implants are typically higher in subjects with SSD compared to those with MHL/CHL [20]. The other non-user with CHL/MHL was unhappy with the sound quality of the device after fitting and remained a non-user, highlighting the importance of patient-centered counseling, rehabilitation, and managing post-operative expectations.

### Audiometric and quality of life benefits

The extended follow-up data confirmed that most patients continue using their Osia System and remain satisfied with the device. Importantly, the clinically relevant improvements in audiometric thresholds (including at higher frequencies), speech reception thresholds in noise, and health-related QoL reported in the previous study [1] remained stable during this 24-month follow-up study. These findings are echoed in an additional study in which Osia System recipients were followed for at least 12 months [21]. Results of the SSQ and HUI questionnaires in this study improved with longer Osia System experience and aided hearing thresholds and speech recognition in quiet were also significantly improved in comparison to the unaided situation [21]. The clinical benefits experienced with the Osia System soon after fitting are therefore expected to remain over the longer term.

### Strengths and limitations

The strength of this study is that it overcomes a previous limitation regarding the paucity of longer term follow-up data with the Osia System. The study was also focused on patient safety and had rigorous safety follow-up, ensuring that all adverse events were captured. Regarding limitations, the study cohort is relatively small, and the number of subjects with SSD prevented a sub-analysis being performed for this subgroup. However, there are published data available demonstrating the safety and efficacy of the Osia System in this population [2]. As seen for subjects with MHL/CHL, we would also expect the benefits observed after short-term observation to remain stable for those with SSD, as observed for a small cohort of subjects with SSD and extended follow-up [21]. Studies are now emerging that compare outcomes for hearing-impaired subjects implanted with active and passive bone-conduction systems [22]. Additional studies with larger patient numbers are required to truly capture the benefits of the system in comparison with other bone-conduction hearing implant systems.

## Conclusion

Extended follow-up data collected as part of this clinical study demonstrate that the Osia 2 System remains a safe and effective treatment for individuals with CHL/MHL or SSD over time. Hearing and patient-reported outcomes remained stable from fitting of the SP to 24 months post-implantation. Importantly, the majority of recipients continued to use and benefit from the daily use of the Osia System.

## Data Availability

Aggregated, anonymized study data will be made available upon reasonable request by contacting the corresponding author.

## Declarations

This clinical study was sponsored by Cochlear Limited, a manufacturer and supplier of implantable hearing devices. ATL and CH are employees of Cochlear Limited, a manufacturer and supplier of implantable hearing devices.
